# Dual response to nest flooding during monsoon in an Indian ant

**DOI:** 10.1038/srep13716

**Published:** 2015-09-08

**Authors:** Swetashree Kolay, Sumana Annagiri

**Affiliations:** 1 Behaviour & Ecology Lab, Department of Biological Sciences, Indian Institute of Science Education and Research, Kolkata, Mohanpur, West Bengal 741246, India

## Abstract

Flooding causes destruction of shelter and disruption of activity in animals occupying subterranean nests. To ensure their survival organisms have evolved various responses to combat this problem. In this study we examine the response of an Indian ant, *Diacamma indicum,* to nest flooding during the monsoon season. Based on characterization of nest location, architecture and the response of these ants to different levels of flooding in their natural habitat as well as in the laboratory, we infer that they exhibit a dual response. On the one hand, the challenges presented by monsoon are dealt with by occupying shallow nests and modifying the entrance with decorations and soil mounds. On the other hand, inundated nests are evacuated and the ants occupy shelters at higher elevations. We conclude that focused studies of the monsoon biology of species that dwell in such climatic conditions may help us appreciate how organisms deal with, and adapt to, extreme seasonal changes.

Animals spend considerable time and resources in building and maintaining their homes as these protect them from environmental adversities. Nonetheless these shelters have to be abandoned sometimes and animals forced to relocate. One of the many factors that cause animals to relocate is flooding; this is especially relevant for the multitude of organisms that occupy subterranean homes^1^,^2^. In eusocial insects such as ants, wasps, bees and termites, the nests not only provide shelter to the adults and storage space for resources such as food but also serve as a platform for performing communal activities^3^. Flooding of their nests can cause drowning of the colony members^4^,^5^,^6^,^7^ and disrupt colony functioning^5^,^8^,^9^. Therefore, adaptations that enable organisms to survive nest flooding have direct implications for their continued propagation.

Nest flooding is a frequent event in the lives of ants that colonize areas such as floodplains, rainforests and intertidal zones. It is known that seasonal flooding generally lowers the species diversity and alters species composition of a region^5^,^10^,^11^,^12^. However, the ant species that inhabit these regions have evolved an array of responses to combat the problem of nest flooding. The responses of these ants can be categorized into behavioural responses, physiological adaptations and architectural modifications. Several ant species such as *Tetraponera* sp., *Cladomyrma* spp. and *Cataulacus muticus* that live in the internodes of bamboo and other plant stems exhibit different forms of bailing behaviour which allows them to remove water from their nests^13^,^14^,^15^. Colony members of *Camponotus anderseni –* which inhabit intertidal zones - prevent nest flooding during high tides by plugging the nest entrance with their heads. *Polyrhachis sokolova* utilise air bubbles trapped in the nest galleries to survive nest flooding. These ants are also capable of tolerating high carbondioxide concentrations that build up inside the nests during flooding^2^. Modification to the nest entrance by construction of levees is seen in some species like *Pheidole* spp., *Acromyrmex landolti*, *Dorymyrmex thorasicus* and *Ectatomma opaciventre*. These levees are effective to varying degrees in preventing entry of flood water into the nests^9^. Colonies of *Harpegnathos saltator* construct complex nests with drainage systems that allow the rainwater to run off to a designated chamber away from adults and brood. In addition, they modify the chamber walls to prevent water seepage^16^. When colonies need to resort to completely abandoning their flooded nests some species show specialized behaviour such as ‘alarm runs’ in *Pheidole* spp.^17^ and rafting behaviour in *Solenopsis invicta*^18^,^19^. These species evacuate flooded nests in an organized manner and, thus, minimize the impact of flooding.

In eastern India, our study area, nest flooding is a threat for a part of the year only. Most of the annual rainfall occurs during 3 to 4 months of the monsoon season. During this time it is common to have 1.2 cm rainfall daily and the ground remains water-logged for several days in stark contrast to other months when there is minimal rainfall. Thus, ants in this region have to evolve responses to meet this challenge only for a particular period of the year while for the rest of the year such adaptations are rendered unnecessary. Unlike this scenario, ants living in intertidal zones or floodplains are likely to face flooding as a persistent problem.

More than 650 species of ants belonging to 87 genera have been reported from different parts of India and more than 70% of them inhabit subterranean nests^20^. For most of these species monsoon is likely to present a challenge in terms of nest inundation. In the current study we explore the response of one such species, *Diacamma indicum,* to nest flooding during the monsoons in the Gangetic plains of India. *D. indicum* is a primitively eusocial ponerine ant reported from the eastern and southern parts of India and Sri Lanka^21^. Their colony size is small and varies from 20–300 monomorphic adults with a single reproductive individual. The colony size remains largely constant throughout the year and there is no drastic reduction in brood content during monsoon^22^. This is contrary to other ant species in which colony sizes and brood production are reduced when conditions are unfavourable^23^,^24^. Colonies of *D. indicum* have been observed to inhabit simple ground nests consisting of a single chamber which is connected to the exterior by a tunnel. In this study we take four different approaches to understand the response of *D. indicum* to the Indian monsoon. Firstly, we characterize the nest locations and entrances across one year in the natural habitat of these ants. Secondly, we examine the responses of these ants to low levels of nest flooding through observations and experiments in the field. Thereafter, we perform experiments in the laboratory with different levels of controlled flooding and observe the response of these ants vis-a-vis changes to nest architecture, nest abandonment and relocation.

## Results

### Field observations

**Figure 1:**
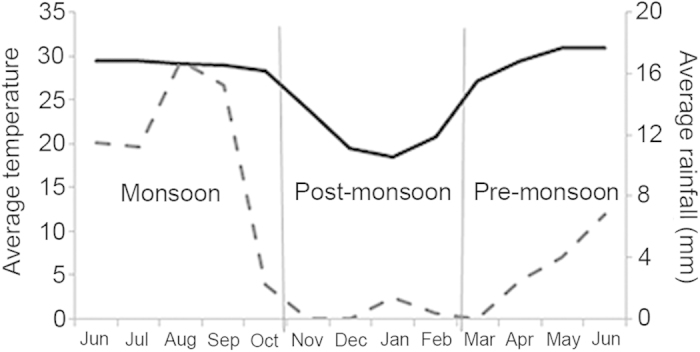
Average monthly temperature (solid black line) and rainfall (dotted grey line) across the three periods of study (pre-monsoon, monsoon and post-monsoon) for 2011–2012 is plotted.

**Figure 2:**
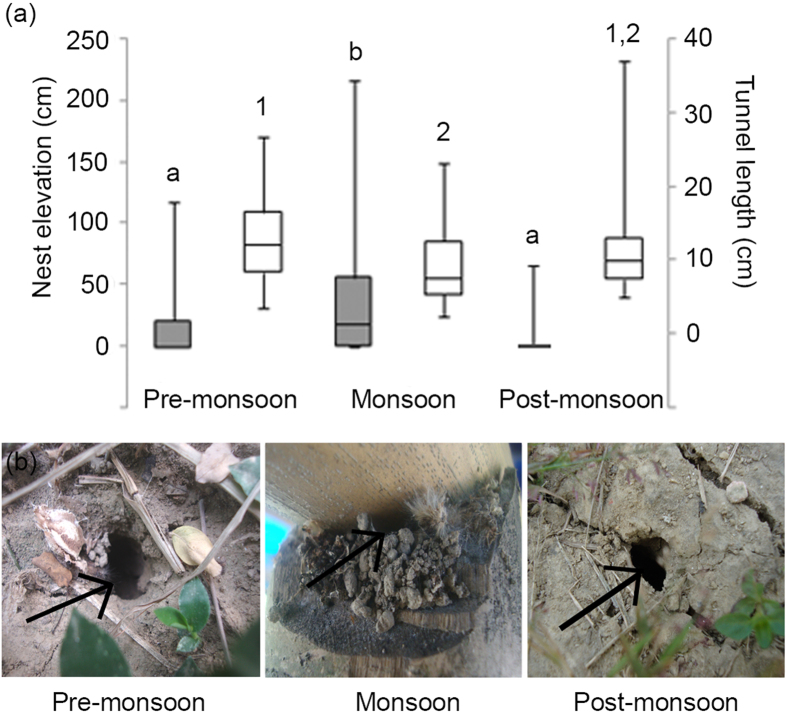
(**a**) Nest elevation (grey bars, primary y axis) and length of entrance tunnel (white bars; secondary axis) of nests studied in the field in pre-monsoon, monsoon and post-monsoon periods. Each box represents the interquartile range, the line inside the box represents the median and the whiskers represent the range of the data. Mann-Whitney U test was used to compare nest parameters between different seasons. Bars carrying different letters or numbers are significantly different from each other. (**b**) Representative picture for nest entrances (arrow pointing towards the entrance) observed in pre-monsoon, monsoon and post-monsoon have been presented.

*Diacamma indicum* nests were studied in their natural habitat in eastern India during three periods, namely pre-monsoon, monsoon and post-monsoon (Fig. 1). Few nests observed during monsoon (25.6%) were subterranean whereas 70.3% nests in pre-monsoon (Contingency χ^2^ test, χ^2^ = 16.0, df = 1, p < 0.001) and 82.8% in post-monsoon (Contingency χ^2^ test, χ^2^ = 22.7, df = 1, p < 0.001) were subterranean. During the monsoon period even though some colonies occupied subterranean nests (25.6%) a majority of the colonies occupied nests located in places such as tree trunks (25.6%), hollows of bamboo stems (18.6%), cracks in brick piles (18.6%), fallen logs (4.7%) and other opportunistic nesting sites (7%). Nest elevations varied across seasons (Kruskal-Wallis test, T = 24.7, df = 2, p = 0.001; Fig. 2a). Nest entrances during monsoon (17.8 cm, 0.3–56.5 cm) were located at a significantly higher elevation than in pre-monsoon (0 cm, 0–21 cm) (Mann-Whitney U test, U = 1099.0, df1 = 42, df2 = 37, p = 0.001) and post-monsoon (0 cm, 0–0 cm) (Mann-Whitney U test, U = 978.5, df1 = 42, df2 = 29, p = 0.001) periods.

Entrance areas of the nests were similar across seasons (Pre-monsoon: 1.1 sq cm, 0.7–1.7 sq cm, Monsoon: 1.2 sq cm, 0.8–1.6 sq cm, Post-monsoon: 0.8 sq cm, 0.5–2.2 sq cm, Kruskal-Wallis test, T = 2.04, df = 2, p = 0.3). However, there were notable variations in other nest parameters studied. There were variations in length of entrance tunnel (Kruskal-Wallis test, T = 8.3, df = 2, p = 0.02; Fig. 2a) and building index (Kruskal-Wallis test, T = 13.7, df = 2, p = 0.001; Fig. 2b) across the year. Tunnel lengths of monsoon (7.8 cm, 5.7–12.6 cm) and pre-monsoon nests (12.1 cm, 8.4–16.6 cm) were significantly different (Mann-Whitney U test, U = 821.5, df1 = 32, df2 = 37, p = 0.005) but post-monsoon nests have tunnel lengths (10 cm, 7.5–13.1 cm) comparable to those in monsoon (Mann-Whitney U test, U = 582.0, df1 = 32, df2 = 29, p = 0.09) and pre-monsoon (Mann-Whitney U test, U = 634.5, df1 = 29, df2 = 37, p = 0.2) periods. Based on modifications made at the nest entrance, each nest was assigned a building index value ranging from 0–5. Building index of monsoon nests (2, 2–4) was significantly higher than both pre-monsoon (2, 2–2) (Mann-Whitney U test, U = 1012.5, df1 = 43, df2 = 37, p = 0.04) and post-monsoon nests (2, 2–2) (Mann-Whitney U test, U = 915.0, df1 = 43, df2 = 29, p = 0.001) whereas building indices of pre-monsoon and post-monsoon nests were comparable (Mann-Whitney U test, U = 619.5, df1 = 29, df2 = 37, p = 0.3). During monsoon 41.9% of nests had mounds of soil around the entrance as compared to 18.9% and 6.9% in pre-monsoon and post-monsoon respectively (Contingency chi square test, χ^2^ = 12.4, df = 2, p = 0.002). On a few occasions ants were seen carrying balls of soil from inside the nest in their mandibles and depositing them in the vicinity of the entrance after showers. In addition, the balls of soil were plastered to form consolidated mounds around some of the nest entrances in monsoon, while such consolidated mounds were absent at other times of the year.

### Low levels of flooding

The response of *D. indicum* colonies to low levels of rainfall was examined in their natural habitat by comparing control nests studied after a dry spell with treatment nests characterized after 2–3 days of rainfall. Mild rainfall (8.6 mm per day) was observed to cause the ants to restructure the entrances of the 43 nests examined while such restructuring was absent in the 33 nests examined in the absence of recent rainfall. Most of the restructuring was in the form of building of soil mounds immediately around the entrance and placement of decorations such as feathers, insect wings, cuticles of earthworms and caterpillars, dead insects, pieces of leaves, twigs and bark around the entrance. The building index of nests studied after rainfall (2, 2–2.5) was significantly higher than that of control nests (2, 1–2) (Mann-Whitney U test, U = 961.5, df1 = 33, df2 = 43, p = 0.008).

The conditions that induce *D. indicum* colonies to make changes to their nest entrances were examined by treating nests with water around the entrance (water_out_) and putting water directly into the nest chamber (water_in_). These were then compared to untreated control nests. The water_out_ treatment nests had a significantly higher building index (2.5, 2–3) than control nests (1, 1–2) (Mann-Whitney U test, U = 59.5, df1 = 8, df2 = 9, p = 0.03). Water stress inside the nest by itself did not elicit a significant change to the nest entrance characters as the building index of water_in_ treatment nests (2, 1–3) was not significantly different from control nests (Mann-Whitney U test, U = 58.5, df1 = 9, df2 = 9, p = 0.1). This indicates that low level of water stress in the vicinity of the nest entrance instigates the colonies to respond by making external changes to their nest entrance.

### Nest architecture

**Figure 3:**
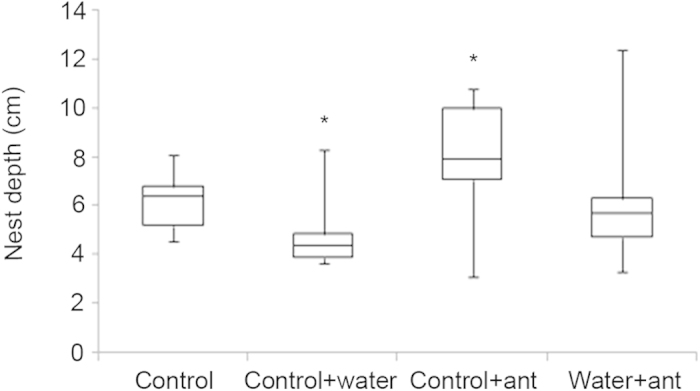
Nest depths measured from casts obtained in the laboratory from four sets of experiments- control, control + water, control + ant and water + ant. Each box represents the interquartile range, the line inside the box represents the median and the whiskers represent the range of the data. Comparisons of parameters were carried out using Mann-Whitney U test and symbols above the boxes indicate categories that are significantly different from each other.

Experiments were performed in the laboratory to study the changes made to the nest structure by *D. indicum* colonies on being subjected to mild water stress. The nest depth obtained by measuring the vertical length of the casts (entrance tunnel + chamber) was significantly different across experiments (Kruskal-Wallis test, T = 11.9, df = 3, p = 0.008; Fig. 3). Although the depths of the nests made in the control + ant treatments (7.9 cm, 7.1–10 cm) were higher than the nest depths in all other treatments, pair-wise comparisons show that the depths of control + ant nests were significantly higher than only control + water nests (4.4 cm, 3.9–4.9 cm) (Mann-Whitney U test, U = 82.5, df1 = 8, df2 = 12, p = 0.03). Depths of control + ant nests were not significantly different from control nests (6.4 cm, 5.2–6.8 cm) (Mann-Whitney U test, U = 76.5, df1 = 8, df2 = 12, p = 0.1) and water + ant nests (5.7 cm, 4.7–6.3 cm) (Mann-Whitney U test, U = 111.5, df1 = 12, df2 = 12, p = 0.1). The depths of nests obtained after water + ant treatment were comparable to control nests (Mann-Whitney U test, U = 56.5, df1 = 8, df2 = 12, p = 0.5) and control + water nests (Mann-Whitney U test, U = 68.0, df1 = 8, df2 = 12, p = 0.3). For pair-wise comparisons of nest depths the corrected p-values using Holm-Bonferroni correction has been presented. Control + ant and water + ant nests had similar number of chambers (Control + ant: 1, 1–1, Water + ant: 1, 1–2, Mann-Whitney U test, U = 82.0, df1 = 12, df2 = 12, p = 0.6) and chamber volumes were comparable between the two treatments (Control + ant: 29.4 cc, 17.6–90.8 cc, Water + ant: 53 cc, 34.6–97.5 cc, Mann-Whitney U test, U = 81.0, df1 = 12, df2 = 12, p = 0.6).

### High levels of flooding

**Figure 4:**
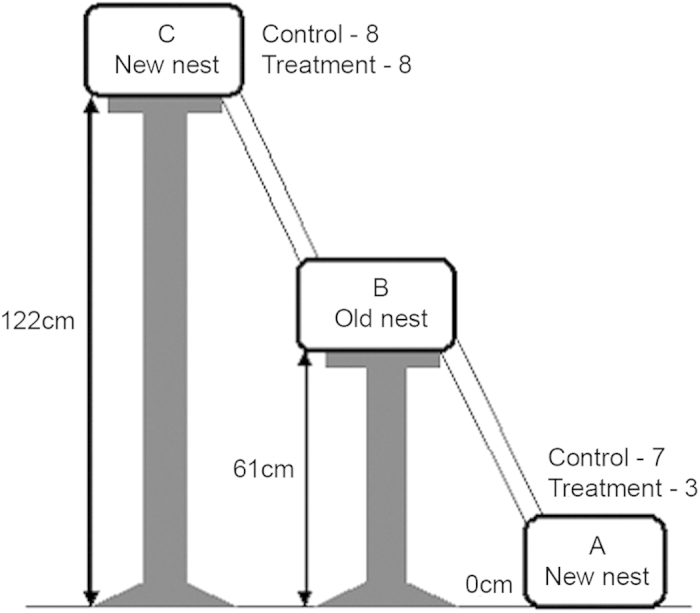
Experimental setup used to evaluate response of *Diacamma indicum* colonies to nest flooding. Three identical boxes lined with plaster of paris and provided with an artificial nest were placed at ground level (**A**) and at elevations of 61 cm (**B**) and 122 cm (**C**) from the ground. A and C were connected to B by 91 cm bridges. The old nest with the colony was placed in B. The figures next to boxes A and C indicate the number of colonies that relocated to the upper and lower nests in control and water treatment experiments.

The response of *D. indicum* to high levels of water stress was examined by placing the colony at a height of 61 cm from ground level (B in Fig. 4) and providing two nests of similar quality at heights of 0 cm and 122 cm (A and C respectively in Fig. 4). Each colony was subjected to a control treatment and water treatment in random order. The colonies had equal probabilities of relocating to either nest on being subjected to stress. During the exploration period, both the new nests were discovered by scouts in 31 out of the total 32 relocations. In the control experiments all the colonies moved out of the old nest and relocated to a new shelter whereas all except one colony relocated out of the flooded old nest following water treatment. Only 1 colony remained split at the end of the observation period of 6 hrs after the removal of the nest cover in the control experiments. In the water treatment experiments, 1 colony remained in the old flooded nest, 2 colonies remained clustered in the bridge just outside the box containing the old nest and 2 remained split at the end of the observation period. As these colonies had not made a final choice of nesting site, they were omitted from the subsequent analysis. In the control experiments 8 colonies moved to the upper box whereas 7 colonies relocated to the lower box (Goodness of fit test, χ^2^ = 0.07, df = 1, p = 0.8). The median time taken to complete the relocations was 210 mins (120–170 mins). In the water treatment experiments 8 moved to the upper box placed at an elevation of 122 cm from the ground while 3 relocated to the box placed at ground level (Goodness of fit test, χ^2^ = 2.3, df = 1, p = 0.1). The median time taken by these colonies to relocate to the new nest was 210 mins (180–270 mins). In both cases qualitative observations indicate that majority of the colony members were tandem run to the new nests.

## Discussion

The nesting dynamics of *Diacamma indicum* and its response to nest flooding caused by rainfall during monsoon have been investigated by a combination of field survey and laboratory based experiments. Our field study reveals that rainfall affects the structure and location of the nests. Nest parameters like elevation and entrance characteristics change across the year with prevailing weather conditions with monsoon having the most profound effect. *D. indicum* colonies exhibit two specific responses to nest flooding during monsoon: (1) Changes are made to the entrances of nests; and (2) subterranean nests are abandoned. In the natural habitat colonies are observed to occupy nests at higher elevations and in the lab experiments there is a tendency to occupy nests at higher elevations.

Significant changes are made at the nest entrance in the form of soil deposition and decorations (fortification) during monsoon presumably as an adaptation to avoid nest flooding in *D. indicum*. Experiments in the field indicate that even low levels of water stress around the nest entrance are enough to stimulate the colonies to make changes to the nest entrance. When only the nest chambers were subjected to water stress without any disturbance at the entrance, colonies did not respond with significant modifications to the entrance. This indicates that entrance modification is a direct response to water stress at the surface of the nest and not a simple consequence of moist soil removal to modify the internal chambers. Although excavated soil in variable amounts was present around nest entrances throughout the year, in monsoon the balls of soil were plastered to form consolidated mounds around the entrances. In some species of ants these mounds are known to function as levees to prevent flood water from entering the nest chamber during shallow inundation^9^. It is possible that *D. indicum* too builds mounds of soil to provide a barrier against nest flooding. Further experiments will have to be conducted to study the formation, structure and effectiveness of these mounds under conditions of low water stress in the beginning of monsoon and high water stress as monsoon progresses. More nests in monsoon had decorations around the entrance than was observed at other times of the year. However, the significance of the decoration items during monsoon is not clear in the case of *D. indicum*. It is known that *Diacamma rugosum* use such decorations to harvest dew drops which meets a substantial part of their water requirement^25^. Experiments involving manipulation of mounds and decorations are required to understand their role in preventing seepage of water into the nest.

Observations in the natural habitat of *D. indicum* show that in the pre-monsoon and post-monsoon periods most of the nests were subterranean while monsoon nests are located at higher elevations. In monsoon the nest locations are more varied such as within fallen logs, hollows of bamboo stems, cavities in tree trunks, brick piles, and cracks in walls, with few subterranean nests. Further experiments in the laboratory confirm that during high levels of flooding the colonies prefer to relocate. Even though 8 out of 11 colonies relocated to an alternate nest at a higher elevation this was not statistically significant. These experiments in the laboratory using high levels of flooding enabled us to show that although ants have a tendency to move to higher elevations, colonies are also likely to relocate to an alternate nest of equal quality if it is available at lower elevations. This indicates that nest flooding does not directly influence the ants to choose nests at higher elevations. It is possible that the unavailability of subterranean nests during monsoon results in colonies seeking shelter at higher elevations. Seeking out these naturally available nests at higher elevations would be a beneficial adaptation enabling the colonies to avoid the problem of constant nest flooding as monsoon progresses.

There are several costs likely to be associated with nest movement. Substantial energy is presumably expended in relocating the colony^26^,^27^, particularly to nests at higher elevations, and in constructing new nesting sites^28^. Colonies face the risk of fragmentation and loss of members during relocation and there is increased risk of predation during movement of reproductives and other colony members to the new nest^8^. Colony activities such as foraging may also be affected as foragers have to climb up with any food items making it more cumbersome and possibly more energy intensive. On the other hand, this strategy ensures survival of the colony by escaping flooded nests during monsoon. Similar vertical migration to escape nest flooding has been observed in other ants^29^ and several other invertebrate species^30^,^31^. In most cases these species are seen to move up tree trunks, sometimes up to the level of the canopy. However, *D. indicum* colonies seem to be more flexible in their nest site selection. The factors that influence the timing of relocation, the choice of nest site, the duration for which these nests are occupied together with nest architecture have to be investigated further to gain a comprehensive understanding of the impact of different seasons on the lives of these ants.

The entrance tunnels of the nests were shorter during monsoon as compared to nests in the other two seasons. Since direct measurement of nest depth was not possible in the natural habitat of these ants, entrance tunnel was used as a proxy for estimating nest depth. Shorter tunnels are suggestive that the nests are shallower during monsoon. Occupying shallow nests during monsoon may have certain advantages. Due to rainfall the soil becomes moist and soggy, thereby, decreasing the structural integrity of nests at greater depths and increasing the risk of structural collapse of tunnels and chamber walls. However, due to the lack of a clear pattern in the casts used to probe internal nest structure, experiments in the laboratory were not conclusive as to whether occupying shallow nests is a direct response to monsoon. All the nests in the laboratory had a single chamber and the chamber volume was comparable between nests of different treatment groups. Nest structure may be further affected by other factors such as colony size and duration of occupancy which have not been considered in the present study.

In conclusion, we have documented a dual response by a single ant species to combat the challenge of nest flooding – modifying the nest entrance with soil mounds and decorations and relocating out of inundated nests to alternate nesting sites. Such adaptations enable the colonies to survive extreme seasonal changes such as heavy rainfall during monsoon. Only a few aspects of the ways in which the lives of these ants are affected by monsoon and their adaptive behaviour have been explored in this study. However, many other features of their life history such as their foraging habits, activity patterns, and social interactions are also likely to be affected during monsoon. Understanding these various aspects by integrating information from different disciplines will enable us to gain a more comprehensive understanding of the monsoon biology of these ants. Our study highlights the need to examine this aspect of the lives of other organisms that face similar challenges due to shifting seasons or a changing climate.

## Methods

### Study area

Nests of *Diacamma indicum* were studied in their natural habitat between June 2011 and June 2012 in Mohanpur, West Bengal, India (22°56′ N, 88°31′ E). The climate in this region is of tropical wet-and-dry type with an average annual temperature of 26.7 °C and total annual rainfall of nearly 200 cm. Weather data recorded at the weather station located at Haringhata Farms, West Bengal, India (22°57′ N, 88°31′ E) during this year is presented in Fig. 1. The study was done in three periods over the course of this year - pre-monsoon, monsoon and post-monsoon. In the current study, pre-monsoon extends from March to the beginning of June when the average daily rainfall is 2 mm and average temperature is 30 °C. Monsoon starts from mid-June and continues up to October. Most of the annual precipitation occurs at this time of the year. The season is characterized by 11.9 mm rainfall per day and average temperature of 28.9 °C. The post-monsoon season from November to February is characterized by low rainfall (0.5 mm per day) and low temperature (20.7 °C).

### Field observations

Information regarding characteristics of *D. indicum* nests was collected across at least 6 field visits during each pre-monsoon (n = 37), monsoon (n = 43) and post-monsoon (n = 29) periods. Nests were discovered by following returning foragers during early morning or late afternoon. Upon discovery of the nest entrance different features of the nests were recorded. The nest elevation - height of the nest entrance from the surrounding ground level - was measured. Any nest with its entrance at the same level as the surrounding ground was considered as a subterranean nest. The diameter of the nest entrance was measured and the area of the entrance was calculated. The length of entrance tunnel was measured by inserting a stick through the entrance till it hit either a bend in the tunnel or the floor of the nest chamber at the end of the tunnel. Most of the entrance tunnels were bare and generally unobstructed making it easy to insert the stick. This entrance tunnel length was used as a proxy to estimate the minimum nest depth in the different periods. The presence or absence of loose soil or a mound of consolidated soil surrounding the nest entrance was noted. Decorations in the form of caterpillar cuticles, feathers and dead insects around the entrance were also noted. The various modifications made at the nest entrance were quantified by using a building index. Building index value ranged from 0–5 as explained below. Nests with bare entrances without any modifications in the soil immediately surrounding the entrance and no decorations received a score of 0, nests with soil mound constructed around the entrance were assigned a value of 3 while nests where the entrance was completely restructured using soil and different decoration items received the highest score of 5 (Fig. 2b). Depending on the number of decorative items and characters of soil mound the intermediate scores were assigned. Further details about the method used to calculate the building index have been included in Supplementary Table S1. The various nest features recorded were compared across different seasons and different treatments to examine the influence of changes in weather conditions across the year.

### Low level of flooding

In order to measure the response of *D. indicum* colonies to low levels of nest flooding, nests (n = 43) were located in Mohanpur, West Bengal, India after a spell of mild showers lasting 3–4 days in May and June 2014. Nest details were collected after 3 such instances when a total of 25.2 mm, 20.5 mm and 48.5 mm of rainfall were recorded. These were then compared to “control” nests (n = 33) which were located in the same area on 3 instances following a dry spell of at least 3–4 days. All nest entrances were characterized and based on the presence or absence of soil mounds and decorations, each nest was assigned a building index value as mentioned earlier.

A further study was done to examine the effects of water only at the entrance and only inside the nest chamber, separately, on the characteristics of the nest entrance. This allowed us to explicitly examine whether water at the entrance was enough to instigate the colonies to make changes at the entrance or changes were made only when the water penetrated into the nest chamber. Nests (n = 26) were identified in Mohanpur, West Bengal, India in March and April 2015 and were subjected to 3 treatments - control, water_in_ and water_out_. In the control treatment, the end of an intravenous tube was carefully inserted into the tunnel as far as it would penetrate and held in position for about 3 minutes after which it was taken out. This was done on 3 consecutive days. This allows us to control for the physical disturbance caused by this intrusion. In the water_in_ treatment, the drip chamber of the intravenous tube was connected to a plastic bottle containing water and the end was inserted into the entrance tunnel as far as possible. By adjusting the roller clamp, water was allowed to flow from the bottle into the nest at a constant rate. Using this method 50 ml of water was poured in 3 minutes directly into the nest without causing any changes outside the nest. This treatment was also performed on 3 consecutive days. In the water_out_ treatment, 200 ml of water was sprinkled in and around the nest entrance on each of 3 consecutive days. On day 4 after the start of the experiment, the nest entrance was characterized as in the previous section. Scoring of the nest entrances was conducted in the blind for all colonies to avoid any potential bias.

### Nest architecture

Plastic containers (diameter 31 cm, depth of 33 cm) were half filled with soil and a 6 cm long plastic tube (1 cm diameter) with a rubber balloon attached at the end was placed in each container with the balloon semi-buried in the soil. The remainder of the container was then filled with soil leaving the top (free end) of the tube exposed. Once in place the balloon was inflated to a fixed volume in order to form a chamber at the end of the tube and the tube was sealed. The soil was then packed in tightly by applying pressure manually and water was poured to ensure that the soil would hold shape. After allowing 4–5 days for the soil to settle and dry out, the balloon was deflated and withdrawn along with the tube thus creating a basic nest with an entrance tunnel and a single chamber. Colonies (n = 24) of *D. indicum* were collected from Mohanpur, West Bengal, India between July and October 2012 and were kept in the laboratory in plastic boxes lined with plaster of paris. Within each box, colonies were housed in a petri plate lined with plaster of paris covered by a watch glass simulating the nest. The colonies were given *ad libitum* food consisting of ant cake, honey, water and termites. In the control + ant experiment (n = 12), the colonies were released in the plastic containers where they occupied the artificial nest we had created. These colonies were left undisturbed for a week with *ad libitum* food and water. In the water + ant experiments (n = 12), after placing the colonies in the containers and their occupation of the artificial nest, 40 ml of water was sprayed each day on 3 consecutive days in and around the nest entrance. No further manipulations were done for the remainder of the week. At the end of the week for each treatment plaster of paris was poured into the nests and the resulting casts were later dug out after the plaster had hardened. Details of the internal structure of the nests such as depth, number of chambers and chamber volume were obtained from the casts. In order to compare the changes made to the nest by the ants in response to water stress with that due to any physical changes caused merely due to passage of time or due to water treatment, two further sets of experiments were performed. In the control treatment (n = 8), the containers with empty nests were left undisturbed for 7 days. In the control + water (n = 8), the container with empty nests received 40 ml of water each day for 3 consecutive days and were then left undisturbed for the remainder of the week. Plaster of paris was poured into the nests, the casts extracted and the same parameters measured.

### High level of flooding

Experiments were done in the laboratory with colonies of *D. indicum* to assess whether relocation to nests at higher elevations was a direct response of the ants to flooding. Colonies (n = 16) were collected from Mohanpur, West Bengal, India between October 2011 and January 2015 and housed in the laboratory as described in the previous section. The box containing the colony was placed on a platform at a height of 61 cm from ground level (box marked B in Fig. 4). Two plastic boxes lined with plaster of paris accommodating artificial nests similar to the box with the colony (B) were placed on platforms at ground level (box A in Fig. 4) and at a height of 122 cm (box C in Fig. 4) from the ground. Boxes A and C were connected to box B by 91 cm long bridges. For a schematic representation of the experimental setup see Fig. 4. Each colony was subjected to two treatments - control and water - in random order with a gap of at least 72 hours between the two treatments. In the control experiment, after allowing the colony to explore the setup for 2 hours, the nest cover was removed and a light source was placed directly above the nest. This left the colony exposed and provoked members to initiate relocation to a new shelter. The number of adults and brood in each nest box was counted at intervals of 30 minutes. This was continued up to 6 hours after the initiation of the experiment. The location of the colony at the end of the observation period was noted as the final position. In case the colony members and brood were dispersed in multiple clumps or at more than one nesting site, it was considered as a split colony because the final nesting site was perhaps undecided. In the water treatment experiment, after two hours of exploration time a light source was placed above the nest and 40 ml of water was sprayed 5 times in box B at intervals of 30 minutes so that the nest as well as the entire box was flooded. This water treatment did not seem to hinder the ants from walking across the box. Even when they were carrying brood in their mandibles they walked without any perceptible difficulty. The nest cover was removed once the nest was empty to prevent the colony from relocating back to the old nest. Observations were conducted in a manner similar to the control experiments. All statistical analyses were carried out using non-parametric tests with statistiXL (Version 1.8). Median and interquartile ranges have been presented unless mentioned otherwise.

## Additional Information

**How to cite this article**: Kolay, S. and Annagiri, S. Dual response to nest flooding during monsoon in an Indian ant. *Sci. Rep.* **5**, 13716; doi: 10.1038/srep13716 (2015).

## Supplementary Material

Supplementary Information
