# Supplementary material for: Dual response to nest flooding during monsoon in an Indian ant
Source: Sci Rep. 2015 Sep 8;5:13716. doi: 10.1038/srep13716 (PMC4562246; doi:10.1038/srep13716)
Supplement: Supplementary Information [file srep13716-s1.pdf]

Dual response to nest flooding during monsoon in an Indian ant

Swetashree Kolay and Sumana Annagiri

Supplementary Table S1

Building index. The table below gives details of the parameters used to score nest entrances characterized in the field. Based on the modifications made at the nest entrance in terms of soil deposition and presence of decorations each nest was assigned a building index value ranging from 0-5. An explanation accompanied by a picture of nest entrance with an arrow pointing towards the entrance is presented for each score.

Score	Entrance characters	
0	Completely bare entrance without any soil or decorations around entrance	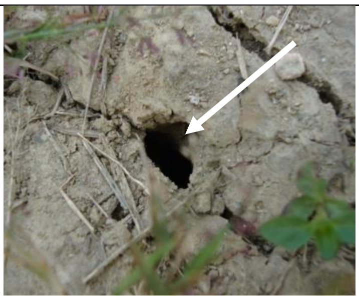
1	Soil balls present in the vicinity of the entrance	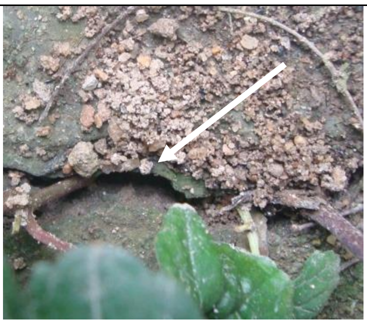
2	Soil balls and decorations are present at the mouth of the entrance in low quantities	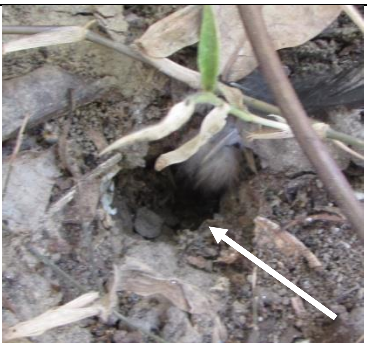

3	Relatively large amounts of unconsolidated mound of soil with or without decorations present around the entrance	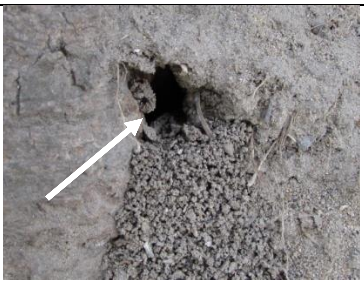
4	Consolidated mound of soil with or without decorations present around the entrance	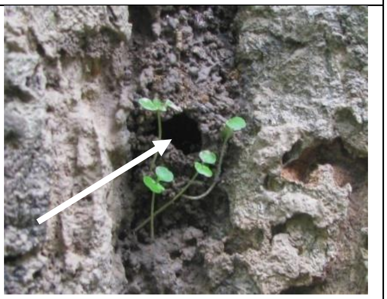
5	Completely restructuring using consolidated soil particles and large number of different kinds of decorations incorporated	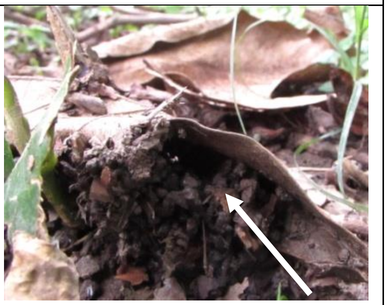

16
